# Emergency response time and pre-hospital trauma survival rate of the national ambulance service, Greater Accra (January – December 2014)

**DOI:** 10.1186/s12873-018-0184-3

**Published:** 2018-10-03

**Authors:** Mohammed-Najeeb Mahama, Ernest Kenu, Delia Akosua Bandoh, Ahmed Nuhu Zakariah

**Affiliations:** 1 National Ambulance Service, Accra, Ghana; 2 Ghana Field Epidemiology and Laboratory Training Program, Department of Epidemiology, School of Public Health, University of Ghana, Accra, Ghana

**Keywords:** Pre-hospital trauma, Ambulance, Survival, Response time

## Abstract

**Background:**

Every year, about 1.2 million people die through road traffic crashes worldwide. Majority of these deaths occur in Africa where most of their emergency medical services are underdeveloped. In 2004, Ghana established the National Ambulance Council to provide timely and efficient pre-hospital emergency medical care to the sick and injured. Pre-hospital emergency medical service is essential for accident victims since it has the potential of saving lives. The study sought to determine the relationship between pre-hospital trauma survival rate and response time to emergencies and factors associated to pre-hospital trauma survival  in Accra, Ghana.

**Methods:**

The study was a cross sectional study which reviewed pre-hospital care forms of trauma patients from the fourteen ambulance stations in the Greater Accra region from January to December 2014. Data were extracted from these forms and the response time estimated. Conscious patients who were alert were categorized as responsive under the AVPU scale. The proportion of patients who survived pre-hospital trauma and  the time pre-hospital trauma cases were responded to was estimated. Multiple logistic regression analysis was conducted to determine which variables were associated with survival.

**Results:**

A total of 652 pre-hospital care forms were reviewed. About 87% survived pre-hospital trauma. The average response time to patients was (16.9 ± 0.7) minutes and the median transportation time of the patient was 82 min. Level of consciousness of a patient and response time of patients transported was found to be significantly associated with pre-hospital trauma survival.

**Conclusion:**

There was a high trauma patient survival rate among victims attended to by an NAS. The average response time in Greater Accra region in the 14 ambulance stations is 16.9 min which is not different from the 17 min recorded in 2013 by NAS. Factors that were associated with pre-hospital survival were alertness in the level of consciousness and response time less than 17 min.

## Background

Each year about 1.2 million people die through road traffic crashes in the world [1]. Many of these deaths could have being prevented if there was adequate pre-hospital medical care worldwide. Africa bears  majority of the deaths and yet with the most underdeveloped emergency medical service (EMS) [2]. The Ghana national road safety 2012 report indicates that 2249 people lost their lives whiles about 14,000 were injured by Road Traffic Crash (RTC) [3]. The Greater Accra region has become infamous for topping the list of cities in Ghana for trauma disaster.

Trauma refers to bodily injury resulting from the application of an external physical force [4]. It ranges from motor vehicle crashes, to burns and blunt assault and is the leading cause of death between the 15 to 45-years age group [5]. Most trauma cases occur in out -of -hospital settings. Road traffic Crash (RTC) is the leading cause of deaths in trauma cases and has unfortunately earned the name “the neglected disease of modern society” since much attention has not been given to it [6].

Pre-hospital Emergency Medical Service (EMS) is the first level of treatment given to patient suffering life threatening conditions by emergency medical technicians (EMTs) and paramedics before they are handed over to the hospital. EMS seeks to reduce response time. That is the time between an emergency call made to an ambulance station and the time a dispatched ambulance gets to the scene for assistance. A shorter response time is known to greatly improve survival [7].

The occurrence of the May 9th Accra sports stadium disaster in 2001 which led to the loss of about 127 lives, provided the leverage for the establishment of the national ambulance service (NAS) in Ghana. The core mandate of the service is to provide timely and efficient emergency medical care to the sick and injured and to transport them to an appropriate facility. The target set for response time in Ghana is eight (8) minutes [8]. Though the total number of ambulance has increased from 24 to 124, the response time target of 8-min has not been achieved. The country’s current response time is 17 min [8].

It has been well documented that shorter response time has the potential of saving lives [9]. However, designing EMS systems to meet these response time targets requires substantial investment which include ambulances, personnel and other resources across the region in other to reduce response time. Work done by Fischer and colleagues in 2000, explains that meeting the set response time target would require substantial cost. [10, 11]. Even though the response time target has not been met. No recent assessment has been made on how Ghana’s response time affects its pre-hospital trauma survival. This study determined the National Ambulance Service’s pre-hospital trauma survival rate, its response time to emergencies attended to the in Greater Accra and factors associated to pre-hospital trauma survival.

## Methods

### Study design

This was a record review of pre-hospital trauma patients’ data for the period January to December 2014. Pre-hospital care forms (PCR forms) of trauma patients responded to by the fourteen (14) Ambulance Stations in the Greater Accra Region [Ridge hospital, Accra Airport, Weija, Tema, Accra City, Ashaiman, Teshie, Atomic, Dodowa, Pantang hospital, Mamprobi, La General hospital, Accra Sports Stadium, and Ada Ambulance Stations] were reviewed and data extracted.

### Study area

The Greater Accra Region occupies a total land surface of 1.4% of the total land area of Ghana. Its capital is Accra, which is also the capital of the Republic of Ghana. This region is the second most populated region in the country after the Ashanti Region. Greater Accra Region has a population of 4,010,054, accounting for 15.4% of Ghana’s total population.

The NAS has fourteen ambulance stations (Accra Airport, Accra city (Makola), Amasaman, Ashaiman, Atomic, Dodowa, La General, LEKMA, Accra Sports Stadium, Ridge, Tema, and Weija) which offers service to 62.5% of the region and record an average of 1358 cases annually. The region was chosen because it has 14 ambulance stations and heavy traffic making it difficult for movement of ambulances to the scene of emergencies.

We reviewed records of all trauma cases in the Greater Accra region transported to various health care facilities by the 14 ambulance stations in 2014. These records were obtained from a PCR forms registry provided by NAS. The study focused on trauma which was defined in the context of road traffic crashes. Trauma was limited to road traffic crashes patients who have been conveyed to various hospitals by the national ambulance service.

### Inclusion criteria

Records of patients who sustained pre-hospital trauma and were directly attended to by the National Ambulance Service as well those who were picked by private or commercial vehicles to clinics and subsequently transported to an appropriate hospital by NAS in the Greater Accra Region were included in the study.

### Exclusion criteria

Data from patients with ineligible or missing records that were difficult to decipher were excluded and all pre-hospital trauma (stable) cases that were transported for diagnostic purposes such as X-ray and CT scan in the region were as well excluded. All the cases that the time of transfer from site of injury to the hospital could not be ascertained were not included in the study.

### Data collection

This data was extracted from the records of operations department of the NAS where Pre-hospital care forms filled by EMTs for each patient they transported are kept. Research assistants were trained to help in the extraction of the data. Stations were also visited to confirm and clarify inconsistent data. This was done by going through their occurrence books at the stations.

Data extracted from the forms were:

- Patient characteristics: age, sex, type of injury
- Time the emergency call was made to the station
- Time the ambulance departed to scene,
- Time the ambulance arrived at scene time,
- Time the ambulance departed the scene to the health facility
- Time the ambulance arrived at the health facility
- The time the patient was handed over to the health facility.

### Data processing and analysis

Data extracted was entered into Microsoft Excel 2010 to estimate the following variables: Response time, transportation time and time spent on scene.

Response time was estimated as the duration between the time a call was made for the emergency and time of arrival of the ambulance at the scene of the emergency.

Transportation time was estimated as the duration between when the call was made to the station and the time the patient was handed over to the facility.

On-scene time was estimated as the duration of time the ambulance spent at the scene of the emergency before transporting the patient to the health facility. That is time of arrival at scene of emergency to time of departure.

Case handling time was estimated as time the patient is taken from the scene to the time the patient is handed over to the facility.

EMTs on the field sort patients according to their degree of injuries and transport those with life threating conditions but salvageable injuries first as it is done in triaging. However, this process is not color coded or recorded on their PCR forms.

Conscious patients that were alert were categorized as responsive under the AVPU scale.

Analysis was done using the Stata Version 13. Response time in minutes was categorized as < 4 vs. ≥4, < 8 vs. ≥8 and < 17 vs. ≥17 min respectively. The proportion of patients who survived pre-hospital trauma as well as the proportion of pre-hospital trauma cases that was responded to within eight minutes was estimated. In line with Eisenerg and colleagues findings of response time between 4 and 8 min increasing the rate of survival for cardiac patients, we determined the four (4) to eight (8) minutes response times [12].

Bivariate analysis was conducted to determine which variables were associated with survival. Chi-squared test of proportions was used to determine the relationship between independent variables (response time categories, on-scene time (< 10 vs. ≥10 min), transportation time (< 60 vs. ≥ 60 min), level of consciousness, type of injuries and age) and dependent variable (pre-hospital trauma survival). Level of significant association was determined at 95% confidence interval and *p*-value of less than 0.05. A multiple logistic regression was conducted to test the association between pre-hospital trauma survival and the significant dependent variables while adjusting for age, and sex.

### Ethical considerations

The study protocol was reviewed and approved by the Ghana Health Service Ethical Review Committee of the Research and Development Division of the Ghana Health Services GHS-ERC: 59/12/15. In addition, permission was obtained from the National Ambulance Service to use their data for the conduct of the study. Patient identifiers such as name and address were not extracted. To assure confidentiality, the forms were kept in locked cabinets and the data entered kept in password protected files on the researcher’s personal computer.

## Results

### Description of data

The 14 ambulance stations of the National Ambulance Service from January to December 2014 transported a total of 20,236 patients. Of these 652 were trauma cases that had all been involved in road traffic crashes.

### Description of patient characteristics

About 70% (435/652) of the trauma cases transported were male. The mean age was (33.0 ± 18.6) years. Almost 66% (415/652) of them were within the age group 15 to 44 years.

The mean response time was (16.9 ± 0.7) minutes, with a range of (1–151) minutes. The median time spent on the scene in managing patients was 17 min with a range of (1–150) minutes. The median transportation time for patients was 82 min with a range of (5–552) minutes.

### Response time and patient characteristics

A total of 8 patients did not survive pre-hospital trauma, only one (12.5%) had a response time of < 8 min. However, no significant difference was found between the patients’ characteristics and response times of eight minutes. (Table 1).

**Table 1:** Characteristics for trauma patients attended to NAS in Greater Accra region, 2014 based on the 8 Minute Response Time Criterion

Variable	Number of trauma patients for each response time N (%)		*P*-value
	< 8 min	> 8 min	
Sex	239	413	0.311
Female	69 (33.8)	135 (66.2)	
Male	170 (38.0)	278 (62.0)	
Age			0.121
> 15	37 (43.6)	48 (56.5)	
15 to 44 years	157 (37.9)	258 (62.2)	
45 to 59 years	18 (26.5)	50 (73.5)	
> 60 years	20 (31.3)	44 (68.8)	
Not stated	7 (35.0)	13 (65.0)	
Body parts injured	221	390	0.887
Head	60 (36.8)	103 (63.2)	
Limbs	77 (38.0)	126 (62.1)	
Multiple trauma	22 (33.3)	44 (62.7)	
Body trunk	16 (30.8)	36 (69.2)	
No obvious injuries	35 (34.7)	66 (65.3)	
Neck injuries	11 (42.3)	15 (57.7)	
Not stated	19 (46.4)	22 (53.6)	
Level of consciousness	200	356	0.211
Alert	179 (37.5)	299 (62.6)	
Verbal	4 (25.0)	12 (75.0)	
Pain	9 (36.0)	16 (64.0)	
Unresponsive	8 (21.6)	29 (78.4)	
Not stated	35 (36.5)	61 (63.5)	
Survival	239	413	0.269
Dead	1 (12.5)	7 (87.5)	
Alive	238 (37.0)	406 (63.3)	

### Pre-hospital trauma survival and patient characteristics

Response time and level of consciousness were found to be associated with pre-hospital survival (*p* < 0.05). Other patient characteristics observed such as transportation time and type of injury were not found to be associated with survival. The other response time categories were not significantly associated with survival (Table 2).

**Table 2:** Bivariate analysis between trauma survival and independent variables of pre-hospital trauma cases attended to in Greater Accra, 2014

Variables	Pre-hospital trauma Survival		*P*-values
	Died	Alive	
Level of consciousness			< 0.0001**
Responsive	3 (0.6)	516 (99.5)	
Unresponsive	5 (13.5)	32 (86.5)	
Response time			0.209
*Based on 4 min*			
< 4 min	0 (0.0)	155 (100)	
> 4 min	8 (1.6)	489 (98.4)	
*Based on 8 min*			0.269
< 8 min	1 (0.4)	238 (99.6)	
> 8 min	7 (1.7)	406 (98.3)	
*Based on 17 min*			0.003*
< 17 min	1 (0.3)	422 (99.8)	
> 17 min	7 (3.1)	222 (97.0)	
Types of injuries			1.00
Obvious	7 (1.8)	502 (98.7)	
Not obvious	1 (1.0)	100 (99.0)	
Time spent on scene^a^			0.183
< 10 min	0 (0.0)	197 (100)	
> 10 min	5 (1.2)	415 (98.8)	
Transportation time^a^			1.00
< 60 min	3 (0.9)	371 (99.2)	
> 60 min	2 (0.8)	235 (99.2)	
Age groups			0.175
> 15	1 (1.2)	84 (98.8)	
15 to 44	3 (0.7)	412 (99.3)	
45 to 59	2 (2.9)	67 (97.1)	
> 60	2 (3.1)	62 (96.9)	

^a^missing data

* - p<0.05, ** - p<0.001

Patients that were conscious had higher odds of pre-hospital survival compared to unconscious patients Table 3. We observed that a response time less than 17 min was associated with survival [aOR = 0.08, 95%CI (0.009–0.59)].

**Table 3:** Logistic Regression Analyses to Model Emergency Response Time as a Predictor for pre-hospital trauma Survival

Variables	Pre-hospital trauma survival		Crude odds ratio (95% Confidence Interval)	Adjusted odds Ratio (95% Confidence Interval)
	Died	Alive		
Level of consciousness				
Responsive	3 (0.6)	516 (99.5)	Ref	Ref
Unresponsive	5 (13.5)	32 (86.5)	26.8 (6.1–117.5)**	37.87 (6.0–239.2)**
Response time based on 17 min				
< 17 min	1 (0.3)	422 (99.8)	0 .08 (0.092–0.61)*	0.08 (0.009–0.59)*
> 17 min	7 (3.1)	222 (97.0)	Ref	Ref
Types of injuries				
Obvious	7 (1.8)	502 (98.7)	1.4 (0.18–63.4)	1.8 (0.16–19.7)
No obvious	1 (1.0)	100 (99.0)	Ref	Ref
Sex				
Male	3 (0.7)	445 (99.3)	0.33 (0.05–2.0)	4.6 (0.69–30.8)
Female	5 (2.0)	200 (98.0)	Ref	
Age	8 (1.3)	625 (98.7)	0.95 (0.92–0.98)	0.78 (0.30–2.03)

* - p<0.05, ** - p<0.001

## Discussion

Our study sought to determine the pre-hospital trauma survival rate and response time to emergencies and its associated factors in the Greater Accra region, Ghana. Our findings revealed that, 98.8% of the cases attended to and transported to various facilities in the Greater Accra Region by NAS survived pre-hospital trauma. According to WHO, timely pre-hospital care can save lives [13]**.** EMTs at the scene of an emergency perform Basic Life Support skills such as controlling bleeding, oxygen administration and splinting which can lead to high patient survival. Lack of Pre-hospital care can lead to death from airway compromise, respiratory failure or uncontrolled bleeding. The common practice of commercial and private vehicles carrying trauma patients from accident scene to hospitals have resulted in the death of some patients. This is because these vehicle were not designed for that purpose and also the drivers lack the needed skills required at the accident scene.

Our study also found the average emergency response time for the region to be 16.9 min. This finding is very similar to the national average of 17 min recorded in NAS, 2013 annual report [14]. In Ghana, response time calculations are still based on estimating the mean response time. However, in many countries, emergency response time is estimated by calculating the proportion of ambulances that were able to respond to cases within 8 min. This is referred as fractal response time. In line with that, a suggested target response time of 8 min for at least 90% of emergency responses has become the guideline for many EMS providers worldwide [7]. This is to prevent extreme values from skewing the mean response time in their direction.

In line with fractile response time, England as at April 2015, had more than 75.6% life threatening cases responded to within 8 min [15]. These higher response times recorded are possibly due the fact that Accra is a city in developing country with a lot of traffic. Driving an ambulance through the traffic-laden city involves a lot of maneuvering. In addition, there are no dedicated emergency lanes for ambulances. These factors eventually increases the response time to emergency. Also, many road users do not obey traffic road regulations or are ignorant of them. These reasons could be contributory factors to the delay in the emergency response time. In order to achieve this national response time target, these situations need to be addressed to improve timely response to medical emergencies.

The median transportation time estimated was 82 min with a range 5 to 552 min. This means that after an incident occurs, it takes an ambulance approximately 82 min to get the case to the hospital. In trauma management, it is believed that the first one-hour after trauma incident is crucial to the patient getting to the emergency department in the hospital in order to improve patient outcome. This principle is usually termed as the golden hour guideline. The NAS is therefore not able to meet the golden hour guideline in trauma cases. Thus morbidity and mortality are likely to increase significantly since the injured person does not receive definitive care within 60 min of injury [16]. In our test of association, transportation time was however not significantly associated with pre-hospital trauma survival (*p* > 0.05). This is similar to a study conducted in Germany in 2012 which also found no significant association between survival advantage for trauma patients with shorter pre-hospital transportation times [17]. Another study in Pakistan assessing the trauma outcomes as a result of hospital transfers and delays in transport time also did not find a significant difference in mortality between patients who were presented within 60 min and those presented after 60 min of response times [18]. Shorter transportation times are therefore essential for the survival of trauma patients. From our study, this was however not found to be significantly associated with survival of trauma patients.

The median time spent on scene managing trauma patients was 17 min with minimum of 1 to maximum of 180 min. The platinum ten-minute guideline recommends that patients should be managed for a maximum of 10 min in trauma situations. Spending more than ten-minutes on the scene can lead to splinting patient to death. It is necessary to initiate transport and continue with treatment on the way to the hospital. The first 10 min is crucial in the management of patients. When the platinum 10 min is violated, it decreases the chances of survival [19]. However, from our study, 10 min spent on scene was not found to be associated with trauma survival (*p* > 0.05).

Our results further suggest that, the level of consciousness is significantly associated with pre-hospital trauma survival. The odds of surviving a pre-hospital trauma increased when a patient was conscious and alert. This confrims the findings of  a study in Germany that concluded that patients with Glasgow Coma score of 3 (unresponsive patients) had a poor outcome and even with treatment only 5% of them had good recovery [20].

Response time of 8-min standard was not also significantly associated with trauma survival in our study (*p* > 0.05). We rather observed 17-min response time to be associated with trauma survival (*p* < 0.05). Since Ghana’s current response time is 17 min, reducing the 17-min response time has the potential of improving pre-hospital survival. Blanchard and colleagues found that after controlling for several important confounders, such as severity of injury, age and sex, the response time standard of within 8 min was not associated with improved survival which supports our findings [6].

### Limitations of the study

Ideally, response time would have been appropriately estimated as the time the accident occurred to the time the ambulance arrived at the scene. However, it was not possible to determine the exact response time, since the EMT was not present at the time of the accidents. We therefore estimated response time as the time between the call being made for the ambulance to the time the ambulance arrived at the scene. Also, distances corresponding to various transport times were not recorded on the PCR forms, which made it virtually impossible for us to factor in an important possible predictor like distance in the study. However, the location of the emergencies was indicated on the form and gave a fair idea of distance.

## Conclusion

There is a high trauma patient’s survival rate among victims of accident attended to by the Ghana National Ambulance Service. The average response time in Greater Accra region in the 14 ambulance stations is 16.9 min which is not different from the 17 min recorded in 2013 by NAS. Factors that were associated with pre-hospital survival were alertness in the level of consciousness and response time less than 17 min. NAS should put in measures to reduce the response time and also put in structures to measure fractal response time. Public education on how to reach an ambulance, what should be done when there is an emergency are some of the efforts that have been made to reduce the response time.

## Abbreviations

CPR: Cardiopulmonary Resuscitation
EMS: Emergency Medical Service
EMT: Emergency Medical Technician
NAS: National Ambulance Service
PCR: Pre-hospital Care forms
RTC: Road Traffic Crashes
WHO: World Health Organization
